# Cardiac Safety of mRNA-based Vaccines in Patients with Systemic Lupus Erythematosus and Lupus-like Disorders with a History of Myocarditis

**DOI:** 10.3390/pathogens11091001

**Published:** 2022-09-01

**Authors:** Giuseppe A. Ramirez, Veronica Batani, Luca Moroni, Giacomo De Luca, Giuseppe Pizzetti, Simone Sala, Giovanni Peretto, Corrado Campochiaro, Emanuel Della-Torre, Enrica P. Bozzolo, Lorenzo Dagna

**Affiliations:** 1 Unit of Immunology, Rheumatology, Allergy and Rare Diseases, IRCCS Ospedale San Raffaele, Milan 20132, Italy; 2 Faculty of Medicine, Università Vita-Salute San Raffaele, Milan 20132, Italy; 3 Faculty of Medicine, Università degli Studi di Verona, Verona 37129, Italy; 4 Unit of Cardiology, IRCCS Ospedale San Raffaele, Milan 20132, Italy

**Keywords:** myocarditis, severe acute respiratory syndrome coronavirus 2, vaccines, systemic lupus erythematosus, adverse event, disease flare

## Abstract

Anti-severe acute respiratory syndrome coronavirus 2 (SARS-CoV-2) vaccines may trigger immune-mediated adverse events, including myocarditis. Evidence of vaccine safety in patients with rheumatic disorders and underlying autoimmune myocarditis is scarce. To address this issue, we studied 13 patients with systemic lupus erythematosus (SLE) and allied conditions with a history of myocarditis and receiving mRNA-based vaccines. Data about general and cardiac laboratory tests, treatment, and disease status were collected during routine consultations before and after the primary vaccination course and after each vaccine dose administration, while myocarditis symptoms were closely monitored. A significant increase in troponin levels from baseline was found after 13 (6–20) days from the first (*p* = 0.046) and 17 (4–29) days after the second dose (*p* = 0.013). Troponin levels progressively decreased within 3 (1–6) months in the absence of typical symptoms or signs of myocarditis. A significant increase in the constitutional domain of the British Isles Lupus Assessment Group (BILAG) index (*p* = 0.046) was observed in SLE patients. However, no patient needed any treatment change. mRNA-based anti-SARS-CoV-2 vaccines can apparently be safely administered to patients with SLE and lupus-like disorders with previous myocarditis despite potential subclinical and transient rises in cardiac damage markers.

## 1. Introduction

Systemic lupus erythematosus (SLE) is a multi-organ autoimmune disease characterised by a relapsing-remitting course. Disease flares can be triggered by infectious agents, including severe acute respiratory syndrome coronavirus 2 (SARS-CoV-2), the aetiological agent of the current COVID-19 pandemic [1,2]. Patients with rheumatic diseases, including SLE, are also at increased risk of COVID-19-related morbidity and mortality [2,3]. Although long-term efficacy data from patients with SLE are lacking, mass vaccination campaigns have radically changed the impact of COVID-19 in the general population and have shown good immunogenicity and safety signals in patients with SLE and other immune-mediated disorders [4,5].

Nonetheless, extensive data collection amidst vaccination campaigns has also revealed the potential of anti-SARS-CoV-2 vaccines to induce immune-mediated adverse events, which may also encompass disease flares in patients with rheumatic disorders [6,7,8]. Vaccine-associated myocarditis is a rare adverse reaction to anti-SARS-CoV-2 vaccines. In particular, the overall risk of myocarditis after anti-SARS-CoV-2 vaccination appears 7–40-fold lower than the risk of myocarditis complicating COVID-19 according to comparative studies [9]. Recent metanalyses have also indicated that post-vaccine myocarditis incidence ranges from 11 to 35 cases per million vaccinees receiving an mRNA-based vaccine [10,11], with higher peaks among younger age groups [12]. The male sex was also reported as a major risk factor for post-vaccine myocarditis [12], although a population-based study suggested that the BNT162b2 vaccine might be associated with higher rates of myocarditis among women [13]. There is a paucity of information about the potential role of other clinical factors, such as previous cardiac disorders, towards the risk of post-vaccine myocarditis [14], although viral triggers may prompt symptom recurrence in patients with a history of myocarditis [15,16].

Notably, cardiovascular complications represent a major cause of morbidity and mortality in patients with SLE and might be promoted by disease-specific pathogenic mechanisms [17,18,19]. Cardiac involvement in SLE can exceed 50% of cases when subclinical features are considered [20]. Myocarditis constitutes an insidious manifestation, occurring overtly in up to 10% of patients with SLE but possibly reaching a 40% prevalence according to post-mortem studies [21,22,23]. Patients with lupus myocarditis are exposed to a high rate of disease- and treatment-related complications and more than one-third are reported to die within 18 months from diagnosis [23,24]. Little is known about the potential factors involved in lupus myocarditis onset and recurrence, although active systemic manifestations and subclinical viral infections/reactivations may constitute accompanying features [25]. There are no data on anti-SARS-CoV-2 vaccine safety in patients with SLE and a history of myocardial lupus involvement.

To address this issue, we performed an observational study in a cohort of well-characterised patients with SLE, lupus-like undifferentiated connective tissue disease (UCTD), and mixed connective tissue disease (MCTD), along with a history of myocarditis and receiving a two-dose-plus-booster course of mRNA-based vaccines in the framework of the Italian anti-SARS-CoV-2 mass vaccination campaign.

## 2. Methods

### 2.1. Patients

From April 2021 to January 2022, we enrolled all patients regularly followed up in the Lupus and Connective Tissue Diseases Clinic of San Raffaele University Hospital, Milan, Italy who met the following inclusion criteria: (a) fulfilment of the 2019 American College of Rheumatology (ACR)/European League Against Rheumatism (EULAR) and/or the 2012 SLE International Collaborating Clinics (SLICC) classification criteria for SLE, the 1999 UCTD criteria, or the MCTD criteria [26,27,28]; (b) previous diagnosis of virus-negative myocarditis based on typical cardiac MRI findings or on endomyocardial biopsy [29]; (c) having received COVID-19 vaccination according to the national vaccination protocols [30,31]. Exclusion criteria encompassed: (a) age < 18 years; (b) refusal to consent to data collection and publication. Consent to data collection and publication was obtained in the framework of the Panimmuno Research protocol, conforming to the Declaration of Helsinki and approved by the San Raffaele Institutional Review Board, with reference code 22/INT/2018.

### 2.2. Connective Tissue Disease Status

Data regarding general laboratory tests (blood counts, complement C3 and C4, anti-double-stranded DNA antibodies (ADNA) and C-reactive protein (CRP) levels) and patients’ daily prednisone (PDN) dose intakes were collected during routine consultations before and after the conclusion of the primary vaccination course (consisting of two doses). We also collected data on patient status at the last follow-up visit available. In patients with SLE, disease activity was evaluated using the Lupus Low Disease Activity State score (LLDAS), the Systemic Lupus Erythematosus Disease Activity Index-2000 (SLEDAI-2K), and the 2004 British Isles Lupus Assessment Group (BILAG) score [32,33,34]. Damage accrual was estimated using the SLE International Collaborating Clinics/American College of Rheumatology (SLICC/ACR) Damage Index (SDI) [35]. A disease flare was defined as the occurrence of any new inflammatory manifestation requiring treatment escalation [36].

### 2.3. Clinical and Laboratory Markers of Myocarditis Activity

In the absence of clear indications on the optimal monitoring of patients with a history of autoimmune myocarditis during the vaccination course, a protocol consisting of the serial monitoring of markers of cardiac damage and inflammation markers (including blood counts, troponin T, pro-brain natriuretic peptide (proBNP) and CRP levels) was agreed by consensus among expert cardiologists and rheumatologists (SS, GPe, GDL, and GAR). Patients were therefore prescribed to check these laboratory parameters at baseline and preferably within three to five days from each vaccine dose administration or at least before the following vaccination dose. Blood sample timing was chosen based on the available evidence, showing cases of myocarditis occurring as early as three days after the first dose and reporting the highest incidence between three and five days after the second dose [37,38]. The same tests were repeated after the booster dose, when done, with the same timing. Additional measurements of troponin were repeated in patients with an elevation of troponin over the upper level of normality, according to the referring physician’s indications. Patients were also asked to contact the referring physician for any symptoms compatible with myocarditis (chest pain, pressure or discomfort, dyspnoea, shortness of breath or pain with breathing, palpitations, syncope). Indications to echocardiography and/or electrocardiography in addition to routine checks were planned only in cases of typical symptoms of myocarditis. Patient status was evaluated during the following routine clinical consultations. We defined myocarditis exacerbation as conforming to the United States Center for Disease Control and Prevention (US-CDC) criteria [39], thus modelling on previous studies addressing COVID vaccine-related myocarditis. Therefore, we considered an exacerbation of myocarditis as the co-occurrence of typical symptoms and elevation of cardiac enzymes or suggestive cardiac alterations at electrocardiography or echocardiography [37].

### 2.4. Statistical Analysis

Statistical analyses were performed with STATACORP Stata^®^ version 15. We used the Mann–Whitney or the Kruskal–Wallis tests to compare continuous variables among two or more groups. Wilcoxon’s matched-pairs signed rank test was used to assess intraindividual variations in continuous variables among distinct timepoints. We used the Spearman test to correlate continuous variables with each other. The chi square test was employed for the comparison of categorical variables between groups. The data are expressed as the median (interquartile range, IQR) unless otherwise specified.

## 3. Results

### 3.1. Patients and Disease Characteristics at Baseline

Within a cohort of 281 patients with SLE, 68 with UCTD, and nine with MCTD actively followed up, we identified a total of 13 patients (seven women and six men) with a history of myocarditis in the context of SLE or lupus-like disorders. Eleven patients had SLE, one had UCTD, and one had MCTD. The median (IQR) age at connective tissue disease onset was 30 (25–34) years, and the median disease duration was 17 (4–22) years. The onset of myocarditis occurred after a median delay of seven years from connective tissue disease onset. Joint (82%), mucocutaneous (64%), and haematological (64%) manifestations were the most prevalent in the study subjects. Five patients had a previous history of renal involvement. At baseline clinical evaluation, all patients were in remission or had low disease activity, with only one patient with SLEDAI-2K >4. Only two patients were taking PDN (2.5 mg/day and 3.75 mg/day, respectively). Seven patients were receiving immunosuppressive treatments with mycophenolate mofetil (n = 6) or azathioprine (n = 1). No patient discontinued immunosuppression to enhance vaccine immunogenicity. Seven patients were taking hydroxychloroquine and three were receiving subcutaneous belimumab (Table 1). All patients were scheduled for vaccination with mRNA-based anti-SARS-CoV-2 vaccines.

### 3.2. Clinical and Laboratory Feature Variations over Time

#### 3.2.1. Disease Activity

The median lag time between baseline and post-vaccine evaluation was 6 (4–7) months. Post-vaccine evaluation occurred after 4 (2–5) months after the second vaccine dose. After the primary vaccination course, no major changes in disease activity were detected and no therapeutic changes (including PDN prescription) were required for any patient. Six patients with SLE (54%) had mild worsening scores in one or more BILAG domains, with no patient showing any new A- or B-grade disease activity status. Specifically, three patients had new C-grade activity in the constitutional domain, two patients had new C-grade activity in the musculoskeletal domain (both reporting arthromyalgia), and one had new C-grade activity in both the constitutional and musculoskeletal domains compared to the baseline evaluation. Globally, only changes in constitutional symptoms reached statistical significance for worsening after vaccination compared to baseline (*p* = 0.046). Variations in the LLDAS and SLEDAI-2K scores across the primary vaccination course were non-significant. Moreover, no significant changes were found in the SDI score (Table 2). Patient status was further re-assessed by direct examination (12/13) or phone call (1/13) after 6 (4–8) months from each patient’s last vaccine administration, corresponding to 8 (6–9) months from the visit following the primary vaccination cycle. Compared to pre-vaccine evaluation, there was no significant change in disease activity in terms of LLDAS, SLEDAI-2K, and BILAG scores. Accordingly, there were no significant treatment changes from baseline evaluation to the end of follow-up. During the last clinical evaluation, all patients were off corticosteroids except for one who was continuing to receive low-dose (3.75 mg/day) prednisone (Table 2).

Regarding general laboratory features, a significant increase in the eosinophil count was observed after the first and second doses compared to pre-vaccination values. Haemoglobin concentrations tended to decrease after each dose and were significantly lower at post-vaccinal evaluation than shortly after Dose 2 of the primary cycle. No significant changes were found in the ADNA titre and complement levels over time (Table 3).

#### 3.2.2. Markers of Myocardial Inflammation

**Figure 1:**
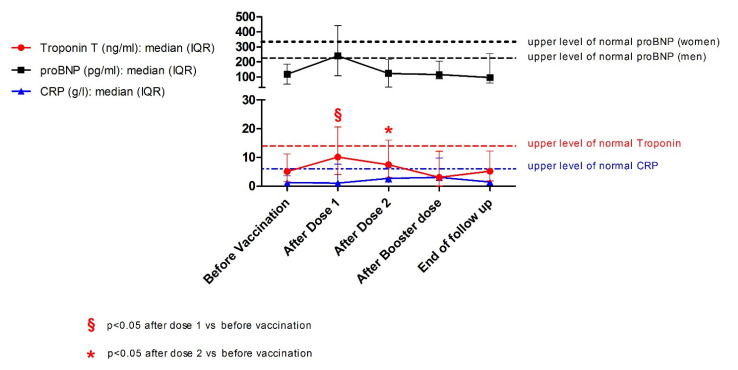
Inflammatory and cardiac-specific marker trends across vaccinations. Line charts showing trends of Troponin (red), proBNP (black), and CRP (blue) levels over time. Each circle, square, or triangle dot represents the median value of each parameter, while whiskers correspond to the interquartile range for each timepoint. Dotted lines indicate the upper limit of normality for each parameter. A significant increase in troponin values, albeit within the normal range, from baseline occurred after the first dose and persisted after the second dose. Abbreviations: CRP: reactive C-protein; IQR: interquartile range.

No patient experienced myocarditis-specific symptoms after vaccination and up to the end of the follow-up. Biomarker measurements were available for six patients after the first dose, 10 after the second dose and at the time of the post-vaccine consultations, five after the third dose, and nine at the end of the follow-up. Details on the timing of the blood sample collection with respect to each dose are reported in Table S1. Two patients (both with SLE) had abnormal troponin levels at baseline. The first patient had a history of severe and slowly resolving myocarditis, with onset three years before vaccination and residual mild elevation of cardiac markers. The second patient had a history of myocarditis occurring 25 years before and had developed post-COVID-19 myocardial infarction three months before baseline evaluation. Troponin levels increased significantly after 13 (6–20) days from the first dose compared to baseline status (*p* = 0.046; N = 6). Three patients (23%; two with SLE and one with MCTD, all males) had troponin levels above the upper limit of normality after Dose 1. Troponin levels after 17 (4–29) days from Dose 2 were also numerically higher than baseline (median variation = 1.0 ng/mL, IQR = 0.6–1.1; *p* = 0.013; N = 10) but lower than after Dose 1 (median variation = −2.0 ng/mL, IQR = −3.2–−0.2; *p* = NS; N = 5). In general, troponin levels progressively decreased over the course of 3 (1–6) months until normalisation or return to baseline levels. Pro-BNP levels mirrored troponin T changes but variations in proBNP concentrations from baseline did not reach statistical significance. No significant changes in CRP levels were observed (Figure 1). Patients with a history of nephritis showed non-significant trends towards wider variations in troponin and proBNP trends after the first two doses (Figure S1). No significant changes in inflammatory or cardiac damage markers were observed at the end of the follow-up compared to baseline status. Details about individual patient trends in cardiac and inflammatory biomarkers are available in the Supplementary Materials (Figures S2–S4). The co-occurrence of increased troponin levels and typical symptoms was not observed. Therefore, no patients met the US-CDC criteria for post-vaccine myocarditis [39]. Heart ultrasound data from routine checks were available for nine patients after the primary vaccination cycle (lag time = 8 (4–9) months) and for five patients at the end of the follow-up (lag time from the last vaccine dose = 2 (2–4) months). No worsening findings were detected.

## 4. Discussion

To determine the tolerability of mRNA-based anti-SARS-CoV-2 vaccines in patients with SLE or similar conditions and myocarditis, we performed a prospective observational study based on serial laboratory and clinical assessments on a cohort of 11 subjects with SLE and two with UCTD and MCTD with lupus-like features. No patient had clinically overt myocarditis recurrence or rheumatic disease flares, and all patients successfully completed their vaccination schedule. In addition, the biomarkers of myocardial injury and systemic inflammation remained within the normal range in the majority of subjects. A statistically but possibly not clinically significant rise in troponin T levels was observed after the first and second vaccine doses. Accordingly, although proBNP levels were numerically higher than baseline after Dose 1, no consistent rise in proBNP was observed over time, indicating that even possible very low-grade inflammatory events following vaccination and involving the myocardium are self-limited and do not affect cardiac function in patients with SLE and similar conditions. A mitigating effect of ongoing immunomodulant/immunosuppressive treatment in this regard also cannot be ruled out. Troponin T variation tended to be wider in patients with a history of lupus nephritis compared to the other patients, consistent with the association between severe SLE and myocarditis [24,25]. Nonetheless, no significant changes in baseline disease activity, besides a mild increase in constitutional symptoms, were observed after vaccination. In this regard, attributing these findings to long-term consequences of vaccination or to expected fluctuations in disease activity is challenging due to the limited sample size of our study and the lack of reference data from the literature, which only reports short-term data [7,31,40]. Longer follow-up studies will probably disclose additional information on the disease and treatment course of these patients over time.

Myocardial inflammation following vaccination is not exclusive to anti-SARS-CoV-2 vaccines, as it has also been reported for a multitude of other vaccines [41]. The pathogenesis of anti-SARS-CoV-2 and non-anti-SARS-CoV-2 vaccine-related myocarditis remains, however, obscure. Intrinsic spike protein cardiotoxicity and cross-reactive autoimmune responses have been proposed as potential pathogenic mechanisms [42,43,44]. Cytotoxicity might also cause an enhanced release of self-antigens, promoting epitope spreading, a key event in autoimmune disease progression and exacerbation [16]. However, in silico studies apparently do not support the hypothesis that the SARS-CoV-2 spike protein sequence overlaps with classical myocarditis-associated autoepitopes [45]. In addition, clinical data suggest that vaccine-induced myocarditis has a very short course, with onset within the first few days from vaccine administration (especially after a second dose) and rapid resolution, which might not be entirely consistent with the occurrence of epitope spreading phenomena. Recent evidence suggests that imbalanced oxidative stress control might be implicated in vaccine-related myocardial inflammation [45,46]. Notably, while weaker responses to oxidative stress are observed in males [47], possibly accounting for the higher rates of vaccine-induced myocarditis cases in men [12], the defective neutralisation of oxidative stressors is also a hallmark of SLE, despite its characteristic female-predominant epidemiological profile [48]. Pathophysiological mechanisms associated with impaired oxidative stress responses appear to be particularly relevant for lupus nephritis and cardiovascular complications of SLE [48,49]. Consistently, we observed that alterations in cardiac biomarkers potentially related to low-grade myocardial inflammation tended to be more prominent in patients with a history of nephritis in our cohort.

Taken together, these data suggest that a history of myocarditis is not associated with myocarditis recurrence in patients with SLE and allied conditions receiving mRNA anti-SARS-CoV-2 vaccines, at least in the short term. This evidence is consistent with the good overall safety profiles of these prophylactic agents in the general population and patients with immune-mediated disorders [50,51] and the relatively benign course of vaccine-associated myocardial inflammation reported in the literature [10,11]. Nonetheless, the consistent detection of a relative rise in troponin levels after each vaccine dose may suggest that subclinical myocardial inflammation actually occurs in patients with connective tissue disorders and a history of myocarditis, possibly confirming the prudential need for closer monitoring of these subjects during their vaccination schedule and later on in their follow-up.

Furthermore, caution is warranted in the interpretation of the data provided in this study, due to multiple potential confounding factors and limitations. First, although representative of the prevalence of myocarditis in SLE and similar conditions, our study cohort had a small sample size. In addition, we had a relatively short timeframe of observation after vaccination, which prevents robust generalisability of our results, especially considering the very-low prevalence of our target event, that is, vaccine-induced myocarditis. Third, we included patients with UCTD and MTCD along with patients with SLE, possibly missing potential disease-specific features associated with anti-SARS-CoV-2 vaccinations against a background of myocarditis. Fourth, the timing of laboratory analyses and clinical assessments was non-homogeneous, due to the data being representative of the “real-life” scenario of the 2021 mass anti-SARS-CoV-2 vaccination campaign in Italy. Fifth, we did not have laboratory and clinical data from a non-at-risk comparator group, and we only tested troponin T instead of troponin I as a marker of cardiac damage. Notwithstanding these limitations, this study constitutes the first description of the safety profile of mRNA-based anti-SARS-CoV-2 vaccines in patients with SLE and lupus-like disorders with a history of myocarditis. In addition, it reports the first application of a protocol for the serial monitoring of patients at potential risk for myocarditis recurrence across the vaccination schedule and characterises trends of cardiac biomarkers over time in patients receiving anti-SARS-CoV-2 vaccines on a background of connective tissue disorders and myocarditis.

## 5. Conclusions

In conclusion, we have shown that mRNA-based anti-SARS-CoV-2 vaccines can apparently be safely administered to patients with SLE and lupus-like disorders with previous myocarditis, despite potential subclinical and transient rises in cardiac damage markers. However, our relatively short follow-up warrants caution in extrapolating this evidence to the long-term, indicating the need for further confirmatory studies.

## Figures and Tables

**Table 1:** Clinical features.

Feature	Value
SLE | UCTD | MCTD: n (%)	11 (85) | 1(8) | 1(8)
Females: n (%)	7 (54)
Age at UCTD/MCTD/SLE onset (years): median (IQR)	30 (25–34)
Age at myocarditis onset (years): median (IQR)	37 (30–40)
Disease duration (years): median (IQR)	17 (4–22)
General clinical features: n (%)	
Joint manifestations	9 (82)
Mucocutaneous manifestations	8 (64)
Haematological manifestations	8 (64)
Lupus nephritis	5 (36)
Neuropsychiatric SLE	3 (18)
Serositis	6 (45)
Constitutional symptoms	4 (27)
Positive ADNA	8 (64)
Positive antiphospholipid antibodies	6 (45)
Treatment features	
Prednisone (any dose): n (%)	2 (15)
Immunosuppressants: n (%)	7 (54)
Mycophenolate mofetil	6 (46)
Azathioprine	1 (8)
Immunomodulants: n (%)	8 (62)
Hydroxychloroquine	7 (54)
Belimumab	3 (23)

**Table 2:** Trends of SLE features across vaccinations.

	Before Vaccination	After Vaccination	End of Follow-Up
Disease status			
Patients in LLDAS: n (%)	9 (82)	8 (80)	8 (80)
SLEDAI-2K: median (IQR)	2 (0–3)	2 (0–4)	1 (0–2)
BILAG constitutional: median (IQR)	0 (0–0)	0 (0–1) *	0 (0–0)
BILAG mucocutaneous: median (IQR)	0 (0–1)	0 (0–1)	0 (0–0)
BILAG musculoskeletal: median (IQR)	0 (0–0)	0 (0–1)	0 (0–0)
BILAG haematological: median (IQR)	0 (0–1)	1 (0–1)	1 (0–1)
BILAG neuropsychiatric: median (IQR)	0 (0–0)	0 (0–0)	0 (0–0)
BILAG cardiorespiratory: median (IQR)	0 (0–0)	0 (0–0)	0 (0–0)
BILAG gastrointestinal: median (IQR)	0 (0–0)	0 (0–0)	0 (0–0)
BILAG ophthalmic: median (IQR)	0 (0–0)	0 (0–0)	0 (0–0)
BILAG renal: median (IQR)	0 (0–0)	0 (0–0)	0 (0–0)
SLICC/ACR damage index: median (IQR)	1 (1–3)	2 (1–3)	2 (1–3)
PDN dose (mg/day): median (IQR)	0 (0–0)	0 (0–0)	0 (0–0)

* *p* < 0.05 compared to the pre-vaccination status by ranked sign test.

**Table 3:** General and SLE-specific laboratory feature trends across vaccinations.

Laboratory Tests	Reference Range	Before Vaccination	After Dose 1	After Dose 2	After Vaccination	After Booster Dose
Timing with respect to each timepoint (days)		NA	13 (6–20)	17 (4–29)	NA	7 (2–15)
Laboratory test results						
Haemoglobin (g/dL): median (IQR)	F: 12–16 M: 14–18	14.2 (13.3–15.1)	13.7 (13.2–14.5)	13.8 (13.4–14.6) ^	13.9 (12.9–14.4) §	14.6 (12.2–14.9)
WBC/microlitre: median (IQR)	4800–10,800	5100 (4400–6200)	5350 (4175–5925)	4450 (3593–5470)	5145 (4150–7325)	3690 (3300–5270)
Neutrophils (%): median (IQR)	40–75	54 (51–60)	61 (51–66)	51 (47–64)	60 (56–66)	51 (50–54)
Lymphocytes (%): median (IQR)	20–50	30 (28–34)	27 (25–33)	29 (23–37)	22 (20–30)	36 (33–37)
Monocytes (%): median (IQR)	2–15	10 (7–10)	9 (9–11)	10 (7–10)	10 (7–10)	10 (10–10)
Eosinophils (%): median (IQR)	1–6	3 (1–3)	2 (2–3) *	3 (1–5) *	3 (1–5)	2 (2–3)
Basophils (%): median (IQR)	0–2	1 (0–1)	0 (0–1)	1 (0–1)	1 (0–1)	0 (0–1)
Platelets × 10^6^/microlitre: median (IQR)	130–400	237 (202–260)	204 (139–222)	195 (179–200)	219 (190–258)	229 (223–254)
Positive ADNA: n (%)	Negative	5 (45)	ND	ND	6 (60)	ND
Complement C3 (g/L): median (IQR)	0.90–1.80	1.04 (0.95–1.09)	ND	ND	1.02 (0.89–1.07)	ND
Complement C4 (g/L): median (IQR)	0.10–0.40	0.18 (0.14–0.20)	ND	ND	0.23 (0.15–0.29)	ND

*: *p* < 0.05 compared to the pre-vaccination status; ^: *p* < 0.05 compared to the status after Dose 1; §: *p* < 0.05 compared to the status after Dose 2 by ranked sign test.

## Data Availability

The data underlying this article will be shared upon reasonable request to the corresponding author.

## Supplementary Materials

The following supporting information can be downloaded at: https://www.mdpi.com/article/10.3390/pathogens11091001/s1, Figure S1: trends of troponin T and proBNP in patients with and without nephritis; Figure S2: individual trends of troponin T; Figure S3: individual trends of proBNP; Figure S4: individual trends of C-reactive protein; Table S1: timing and availability of biomarker data.
