# Supplementary material for: Cardiac Safety of mRNA-based Vaccines in Patients with Systemic Lupus Erythematosus and Lupus-like Disorders with a History of Myocarditis
Source: Pathogens. 2022 Sep 1;11(9):1001. doi: 10.3390/pathogens11091001 (PMC9502100; doi:10.3390/pathogens11091001)
Supplement: Supplementary file 1 [file pathogens-11-01001-s001.zip › pathogens-1829946-supplementary.pdf]

Supplementary materials

Table S1. Timing and availability of biomarker data.

	**Baseline**	**After dose 1**	**After dose 2**	**After Vaccination**	**After dose 3**	**End of follow up**
Number of subjects	13	6	10	10	5	9
Timing from the last vaccine dose (days)						
Patient #1	NA	ND	4	NA	17	227
Patient #2	NA	7	46	NA	2	151
Patient #3	NA	ND	3	NA	7	49
Patient #4	NA	18	17	NA	ND	55
Patient #5	NA	5	5	NA	2	ND
Patient #6	NA	29	ND	NA	ND	ND
Patient #7	NA	ND	3	NA	ND	86
Patient #8	NA	2	ND	NA	ND	249
Patient #9	NA	ND	2	NA	ND	ND
Patient #10	NA	21	20	NA	ND	126
Patient #11	NA	ND	38	NA	ND	168
Patient #12	NA	ND	ND	NA	ND	ND
Patient #13	NA	ND	17	NA	15	47
Timing from the last vaccine dose (days): median (IQR)	NA	13 (6-20)	17 (4-29)	NA	7 (2-15)	86 (55-168)
Within one week	NA	3/6	5/11	NA	3/5	0/9
Within one month	NA	6/6	8/11	NA	4/5	0/9
Before the following timepoint	NA	6/6	11/11	NA	5/5	NA

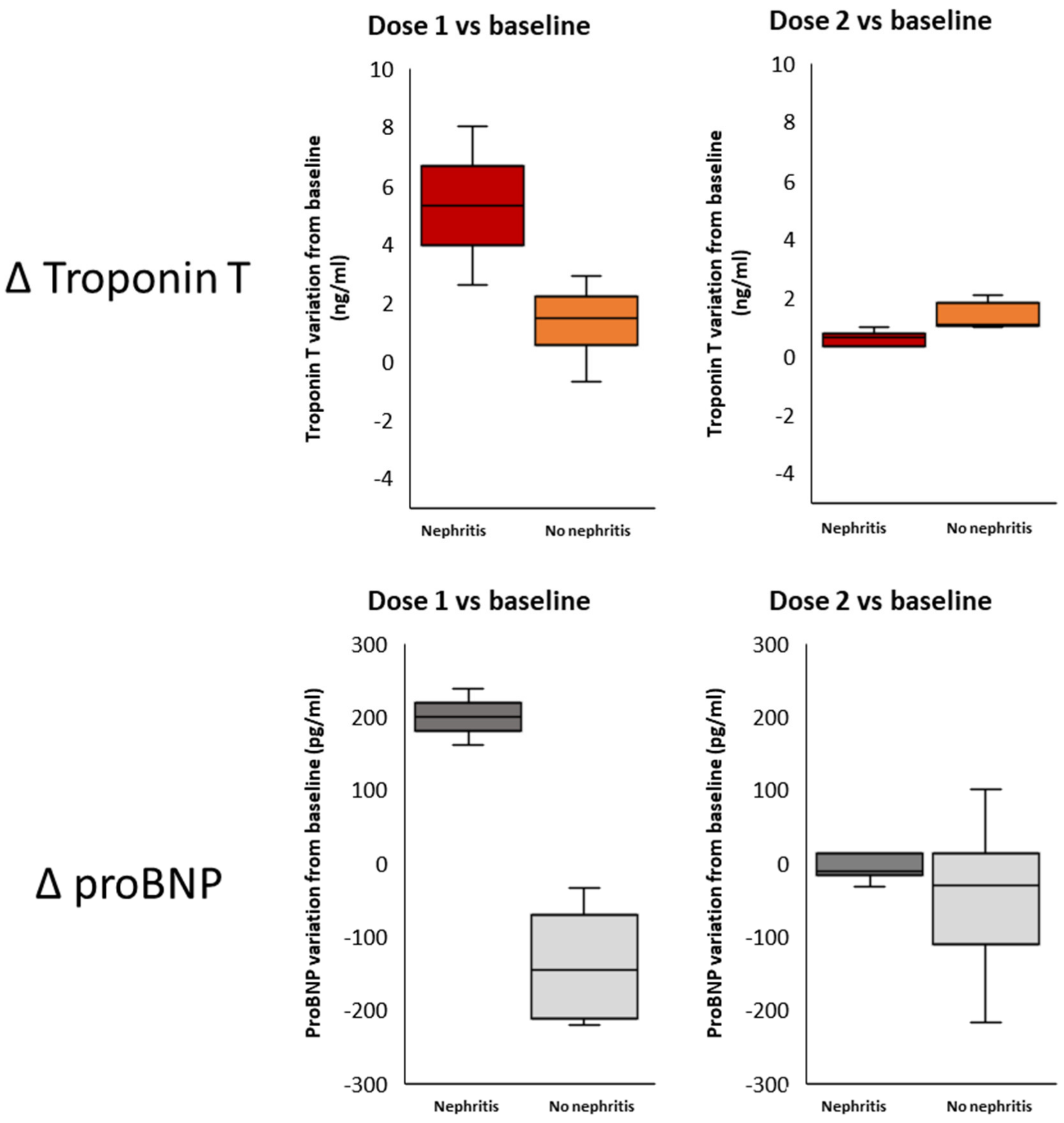

Figure S1. Trends of troponin T and proBNP in patients with and without nephritis.
Boxplots showing the differences in troponin T (upper quadrants) and proBNP (lower quadrants) trends in patients with vs without a history of nephritis. Variations in troponin T and proBNP were calculated as the differences between troponin T and proBNP values after dose 1 (left quadrants) and baseline and between values after dose 2 and baseline (right quadrants).

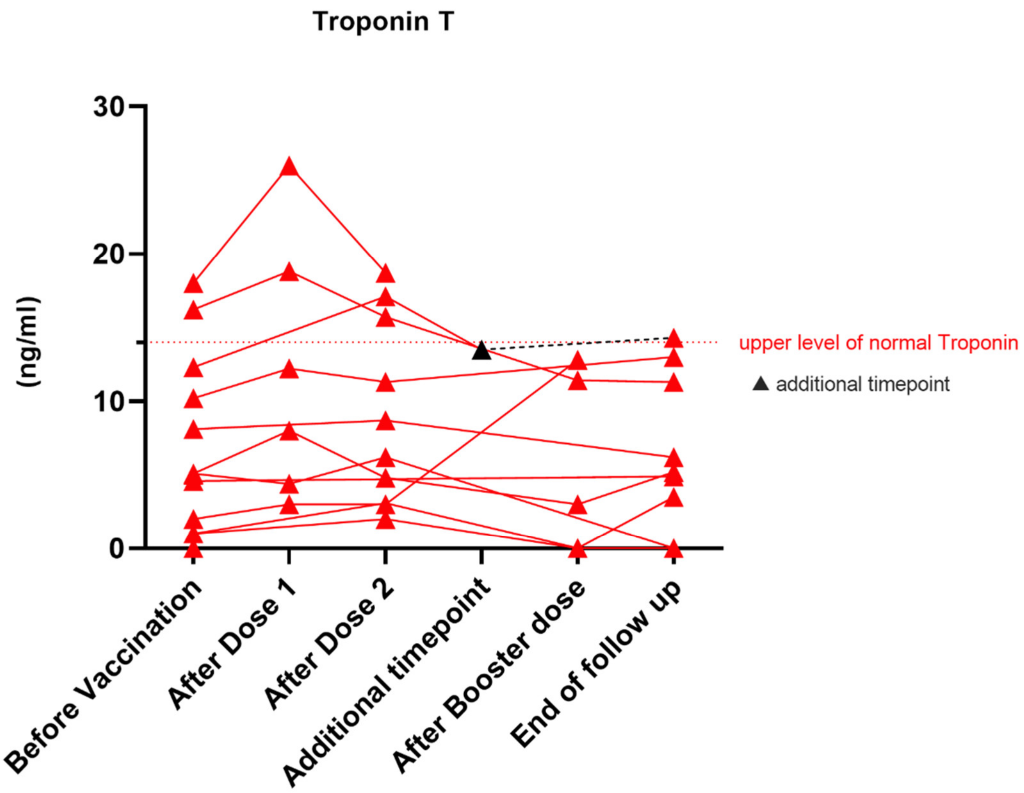

Figure S2. Individual trends of troponin T.

Line charts showing trends of Troponin levels over time. Dotted lines indicate the upper limit of normality.

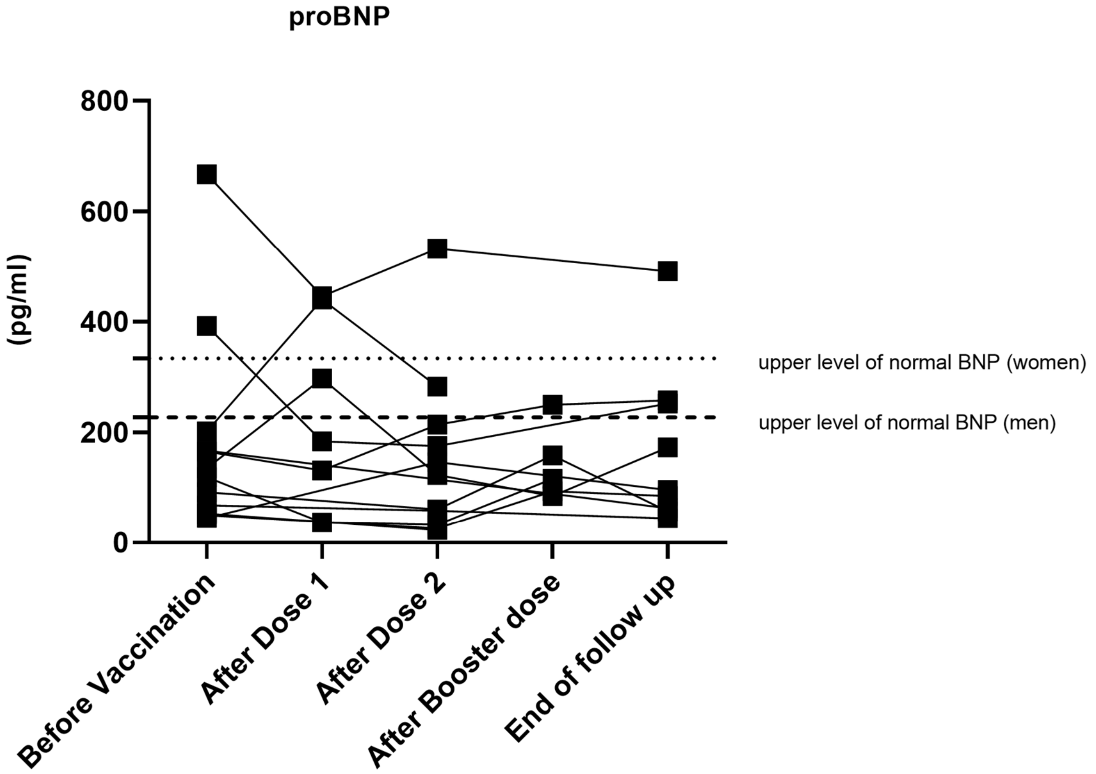

Figure S3. Individual trends of proBNP.

Line charts showing trends of proBNP levels over time. Dotted lines indicate the upper limits of normality in women and men.

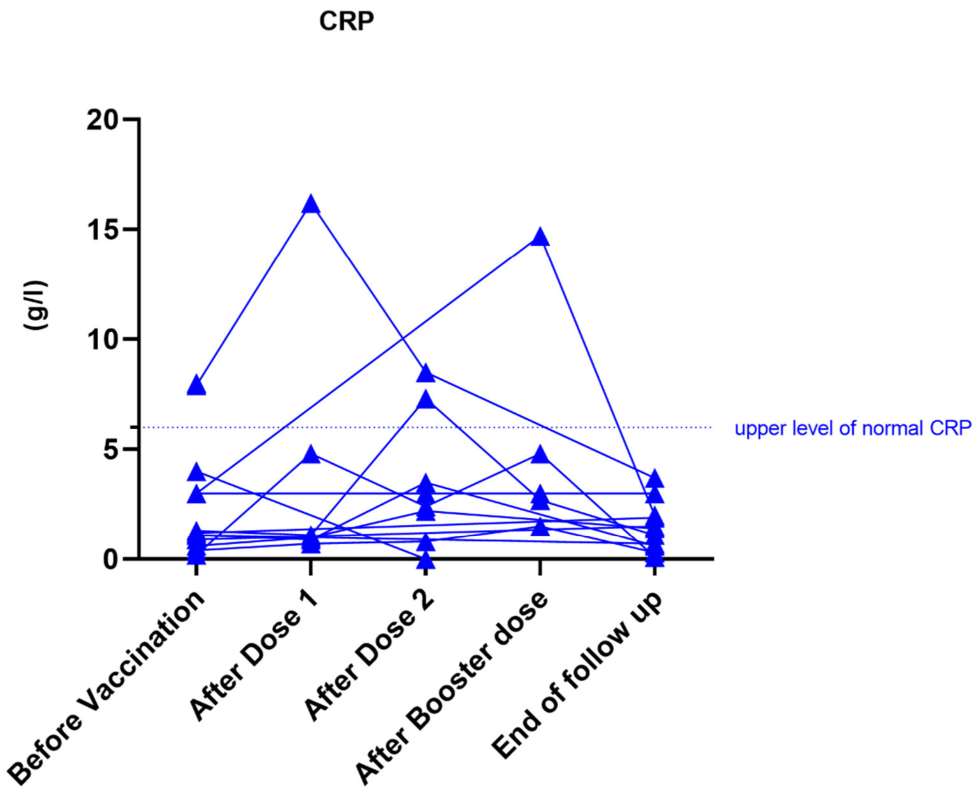

Figure S4. Individual trends of C-reactive protein.

Line charts showing trends of C-reactive protein levels over time. Dotted lines indicate the upper limit of normality.
